# Attentional responses in toddlers: A protocol for assessing the impact of a robotic animated animal and a real dog

**DOI:** 10.1371/journal.pone.0327389

**Published:** 2025-07-02

**Authors:** Mireya Zapata, Carlos Ramos-Galarza, Kevin Valencia-Aragón, Jorge Alvarez-Tello

**Affiliations:** 1 Centro de Investigación en Mecatrónica y Sistemas Interactivos (MIST), Ingeniería Industrial, Universidad Tecnológica Indoamérica, Machala y Sabanilla, Quito, Ecuador; 2 Facultad de Psicología, Pontificia Universidad Católica del Ecuador, Quito, Ecuador; 3 Dirección de Innovación y Vinculación, Escuela Politécnica Nacional, Quito, Ecuador; 4 Centro de Innovación Social y Desarrollo, Quito, Ecuador; Neighborhood Physical Therapy, UNITED STATES OF AMERICA

## Abstract

**Background:** Attentional processes in toddlers are characterized by a state of alertness in which they focus their waking state for short periods. It is essential to develop assessment and attention stimulation protocols from an early age to improve this cognitive function and detect possible deficits in the early stages of cognitive development. **Methods:** This protocol-based article presents a technological approach to assessing the impact of two animated stimuli on toddlers’ attention. The protocol involves presenting a robotic and a real dog to evaluate attentional activation in early development. This dual-stimulus framework may also inform therapeutic and educational programs aimed at fostering cognitive growth in early childhood. **Discussion:** This protocol contributes to assessing physiological attentional responses in toddlers, offering insights into attention evaluation and stimulation during early cognitive development. By incorporating both a robotic and a live dog, it enables the exploration of individual differences in attentional engagement and identifies which stimulus elicits a stronger response.

## Introduction

The human brain is a fascinating organ that not only develops genetically but also shapes itself through constant interaction with the environment [1]. From the earliest stages of life, sensory, emotional, and social experiences influence its structure and functioning, allowing the individual to acquire skills, knowledge, and adaptations that enable them to navigate their context [2,3]. This formation process is dynamic and continues throughout life, making the brain an ever-evolving entity [4].

In the first stage of life, the human brain experiences accelerated development and greatly benefits from interaction with the environment due to its high plasticity. During this period, neural connections form rapidly, allowing the brain to adapt and learn efficiently from sensory, emotional, and social experiences [5,6]. This brain plasticity, which is most pronounced in the early years of life, facilitates the acquisition of fundamental skills such as language, motor control, and emotional regulation, laying the groundwork for cognitive development throughout life [9].

Attention is a cerebral process whose stimulation and development begin at birth. From the very first day of life, brainstem functions involved in arousal and wakefulness begin to develop, forming the foundation for sustained attention in later stages. As toddler grows, they begin to perceive environmental stimuli and gradually develop selective attention, progressively increasing the duration for which they can engage in a task. These milestones are made possible by the ongoing maturation and myelination of various structures within the nervous system involved in attentional processes. As the child continues to mature, they become capable of responding to increasingly complex stimuli (e.g., classroom demands). They also develop the ability to manage interference, sequentially process multiple components within a task, and, most importantly, attend to the complex cognitive functions essential for human development, including reading, writing, and mathematical reasoning [7,8].

This developmental progression is particularly crucial during the first three years of life, when attention manifests through two interdependent yet distinct levels [10]. The arousal level, governing alertness and environmental responsiveness, provides the foundation for curiosity and exploration [11], while focused attention enables task-specific concentration and information retention [12,13] By providing rich and varied experiences that engage both arousal and focused attention, can significantly enhance toddlers’ environmental interactions and subsequent cognitive development [14–16]. In summary, attention is a vital cognitive function that permeates all areas of human life, contributing decisively to individual development and success in every context [17].

During early childhood, many attentional processes are shaped by interactions with stimuli that toddlers perceive as meaningful or engaging. Researchers have shown that toddlers are particularly drawn to stimuli that exhibit animacy—objects that appear alive or capable of intentional action [18]. This animacy can be attributed to anything that shows signs of movement or intentionality, such as robots or living beings like animals, which toddlers naturally associate with purposeful behavior and emotion. In this sense, social robotics plays a valuable role in developmental interventions by displaying signs of animacy, allowing robots to engage toddlers in ways similar to living beings [19]. By providing engaging, interactive stimuli, social robots can effectively capture and sustain toddler’s attention, enhancing learning experiences and promoting both arousal and focused attention—key elements for cognitive development [20]. In the first level of attention, which unfolds during the earliest stages of human development and is the focus of this protocol, we find attention related to arousal or immediate focal response to changes in brain tone and alertness [21]. At this initial level, attentional responses can be assessed by changes in body temperature, motor movements, shifts in gaze toward objects of interest, verbal responses indicating interest in environmental stimuli, somatic emotional responses on the face, and physiological changes in respiration, blood flow, and electrical brain activity [22]. Therefore, this protocol aims to identify the basic physiological changes that occur in toddlers in response to various stimuli, guiding future studies to create stimulation activities that promote attention in the early stages of development.

During early childhood, it is crucial to focus on two key aspects of attention functioning. First, the proper stimulation of this cognitive function is essential to promote its development and maturation, which, in turn, significantly improves toddler’s performance in the learning process [23]. Attention exercised from an early age allows toddlers to acquire the cognitive and emotional skills that are critical for their academic performance and adaptation to new educational environments [24].

Second, the early detection of possible attention difficulties is indispensable for timely interventions. Identifying issues like attention deficit early on enables the implementation of therapeutic and educational strategies that can mitigate the impact of these disorders in later stages of development [25]. Early intervention not only facilitates better adaptation during childhood but also reduces the risk of these difficulties negatively affecting the toddler’s emotional, social, and academic well-being in the future. In summary, both the stimulation and early intervention in attention during the first years of life are essential pillars for a healthy and holistic development [26].

In the context of early cognitive development, selective attention emerges as a pivotal function that enables toddlers to focus on relevant stimuli while inhibiting responses to distractions. This ability, often associated with perceptual inhibition, plays a foundational role in the development of executive functions during early childhood. Studies have shown that during this critical developmental window, toddlers begin to exhibit the capacity to filter competing sensory inputs, a skill essential for the acquisition of self-regulation, goal-directed behavior, and later academic success [27,28]. Given its significance, integrating the assessment of selective attention and underlying inhibitory processes into early evaluation protocols offers a more comprehensive understanding of attentional functioning. This approach aligns with the growing body of research emphasizing the importance of assessing and stimulating executive function-related abilities—such as attention control and inhibitory mechanisms—from the earliest stages of life.

The protocol presented in this research article focuses precisely on the two key aspects mentioned: attention stimulation and early detection of attention difficulties in toddlers during early childhood. To address these aspects, the application of a methodology using concepts of animacy is proposed, utilizing both an animal-like robot and a real dog. Using specialized sensors placed on the hands and feet, the goal is to analyze in detail toddler’s attentional responses to a variety of sensory stimuli. This multisensory approach allows for a more accurate capture of motor and cognitive reactions that reflect the degree of sustained and selective attention [19].

Additionally, a state-of-the-art camera is used to record and analyze somatic markers, such as changes in posture, micro facial expressions, and fluctuations in pupil dilation, which are associated with focused attentional functioning and arousal levels. These physiological markers provide a more complete picture of the toddler’s attentional state, allowing for more precise identification of moments when concentration fluctuates or is interrupted.

The data obtained from these devices will not only enable the development of more effective activities for attention stimulation in toddlers but will also serve as diagnostic tools for the early identification of potential attention deficits. This opens the door to more personalized and timely interventions, significantly contributing to toddler’s cognitive and emotional development. In summary, this innovative protocol offers a comprehensive approach to improving both the stimulation and evaluation of attention during the early years of life.

## Methods

### Research design

This section outlines the design and structure of the research, highlighting the methodological approach employed to evaluate the proposed interventions and their impact. The study utilizes a systematic framework to ensure a robust analysis of the outcomes, with an emphasis on measuring changes over time and establishing reliable causal relationships. Additionally, the characteristics of the participants are discussed, focusing on the selection criteria and ethical considerations necessary for conducting research with toddlers. These considerations ensure a safe and engaging environment for participants, while also maintaining the scientific rigor required for reliable and generalizable results.

### Ethics and dissemination

This research has received approval from the Human Research Ethics Committee of Indoamerica University in Ecuador, ensuring compliance with the highest ethical standards in research involving human subjects. All activities conducted within the framework of this study will be designed to respect the dignity and rights of participants, ensuring that their physical and psychological integrity is safeguarded at all times. In the case of minors participating in the study, informed consent will be obtained from their parents or legal guardians, who will be duly informed about the study’s objectives, procedures, and potential risks. Consent will be requested both verbally and in writing to ensure clear understanding and agreement from all parties involved. Furthermore, parents will be present during all stages of the research, which will not only guarantee the supervision of the minors but also foster an atmosphere of trust and safety. This attention to ethical aspects will be fundamental, not only to protect the participants but also to ensure the validity and integrity of the obtained results, thereby contributing to the credibility and acceptance of the research within the scientific community and society at large. Additionally, the commitment to ethical practices will reinforce the social responsibility of the university and the research team, promoting a respectful and sensitive approach to the participation of toddlers and their families in scientific studies.

#### Type of study

The study will employ a quantitative experimental research design, utilizing pre- and post-test measurements to evaluate the effects of the proposed interventions. The research will adopt a longitudinal approach, allowing for continuous monitoring of the participants over time and observing changes in their attention levels following the implementation of the technological activities. This design is particularly valuable in the field of child development research, as it provides a more comprehensive view of the evolution of attentional capacities at different stages of the intervention.

Experiments will be conducted at various points throughout the study to assess the effectiveness of the activities specifically designed to enhance attention in toddlers. These activities will be based on the use of innovative tools such as sensors and a recognition camera, which will enable precise and objective measurements of the toddler’s attentional responses. Video and sensor data for each intervention are recorded in real time and stored for subsequent analysis. To minimize bias during evaluation, collected data will be anonymized, and evaluators will remain blinded to experimental conditions or any details that could influence interpretation. Each intervention will undergo rigorous analysis to verify its effectiveness, and control groups will be used to ensure that the observed results can be directly attributed to the proposed activities.

To systematically evaluate the attentional responses, several measurement variables will be analyzed, as summarized in Table 1. These variables encompass physiological, behavioral, and interaction-based metrics, providing a comprehensive assessment of how toddlers engage with the experimental stimuli.

**Table 1:** Measurement criteria.

Measurement Variable	Description
Arousal Level	Assessed through physiological responses such as changes in respiration, heart rate, and skin conductance.
Focused Attention	Evaluated by tracking gaze duration and fixation points using an eye-tracking system.
Motor Activity	Measured using accelerometer sensors placed on the toddler’s limbs to detect movement patterns.
Facial Expressions	Analyzed through real-time facial recognition algorithms to detect emotional responses.
Interaction Duration	Time spent interacting with the robotic dog and the real dog, recorded by video.

In addition to the pre- and post-test measurements, periodic evaluations will be conducted throughout the study to identify potential gradual changes in the participants’ attention. This longitudinal approach will not only help to identify the immediate effects of the interventions but also assess their sustained impact over time. Moreover, the use of an experimental design will allow for the establishment of causal relationships between the technological activities and improvements in the toddler’s attention, thus contributing to a better understanding of how technological tools can be effectively used in child development.

Finally, the study’s design ensures rigorous control of variables, which will allow for the collection of reliable and generalizable results. This will provide solid empirical evidence that can be used to guide future research, as well as the development of educational and therapeutic interventions aimed at improving attention in toddlers.

#### Characteristics of participants

The study participants will be toddlers, specifically between 6 months and 3 years of age. This age range has been selected due to the critical importance of attentional development during the first years of life, a key stage in the acquisition of fundamental cognitive and motor skills. All infants participating in the study will fall within typical developmental parameters for their age and will not present any physical, sensory, or cognitive disabilities that could interfere with the evaluation of the proposed activities.

To ensure the protection and well-being of the toddlers, written informed consent will be obtained from the parents or legal guardians of all participants. This consent will be accompanied by a detailed explanation of the study’s objectives, procedures, and any potential benefits or risks, ensuring that parents fully understand the nature of the research and their children’s participation. Additionally, mechanisms will be in place to allow parents to withdraw their children from the study at any time, without any negative repercussions.

Throughout the research process, a rigorous ethical approach will be maintained, adhering to international guidelines for the participation of minors in scientific studies. Procedures will be non-invasive and sensitive to the age and needs of the infants. The experiments will be designed to ensure that the toddlers feel comfortable at all times, using playful and engaging methods that encourage voluntary and spontaneous participation.

Furthermore, Table 2 shows the strict selection criteria that will be implemented to ensure that all participants meet the necessary conditions to guarantee the validity of the results obtained. This careful approach not only contributes to the scientific rigor of the study but also helps to create a safe and protective environment for the toddlers and their families.

**Table 2:** Selection and exclusion criteria for study participants.

	Criterion	Description
**Selection**	**Age range**	**Toddlers between 6 months and 3 years old.**
	**Developmental status**	Participants must fall within typical developmental parameters for their age.
	**Absence of disabilities**	Infants with physical, sensory, or cognitive disabilities that could interfere with the evaluation will be excluded.
**Exclusion**	**Medical conditions**	**Infants diagnosed with neurological, psychiatric, or metabolic disorders, and animal allergies.**
	**Prematurity**	Infants born before 37 weeks of gestation.
	**Developmental delays**	Any diagnosed or suspected delay in cognitive, motor, or sensory development.
	**Parental refusal**	Parents who decline to provide informed consent or withdraw participation.

We expect recruitment of participants to be finalized by July 2025, data collection to conclude by August 2025, and preliminary results to be available by October 2025.

## Materials and equipment

**Fig 1:**
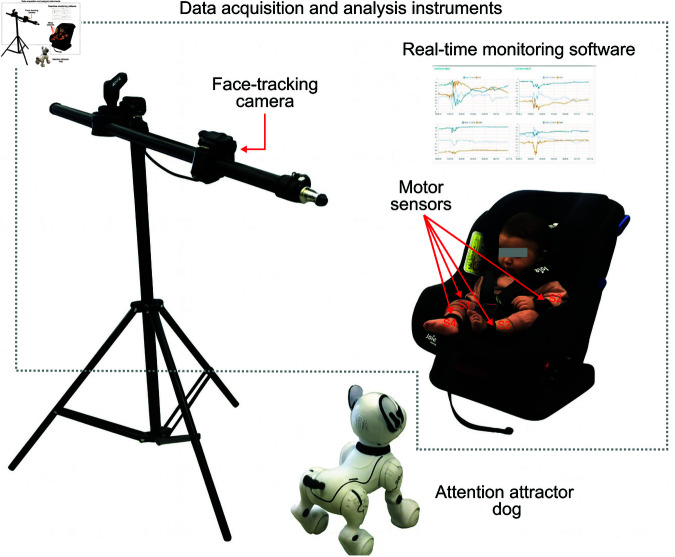
Experimental setup. In this intervention scenario, the infant is seated in front of an attention-attracting dog (either a real dog or a robotic dog). S1 to S4 are accelerometer sensors that measure the infant’s reaction to both stimuli.

This section provides an overview of the primary components involved in the analysis of toddlers interactions, detailing their specific roles within the experimental setup. The configuration incorporates an attention attractor dog, as well as data acquisition and analysis instruments, all selected to ensure precise measurement while maintaining the safety and comfort of the participants. Fig 1 illustrates the materials and equipment utilized in the experimental setup.

### Attention attractor dog

This part of the study focuses on using an animated attractor dog, either a real dog or a robotic dog. Both will be incorporated into the interaction activities, and their role in engaging the toddler’s attention will be examined. The following sections will describe the specific characteristics and selection criteria for each type of dog.

#### Real dog

For the animal interaction activities, a dog will be meticulously selected based on its familiarity with close human companionship and its demonstrated docile, friendly, and stable behavior. This approach will serve to minimize any potential risks, particularly for toddlers, who may be more susceptible to feeling intimidated or uncomfortable around larger animals or those with unpredictable temperaments. Proper socialization of the dog is a fundamental requirement; it must be accustomed not only to interacting with people but also to doing so calmly in various environments and situations, without displaying signs of aggression, nervousness, or anxiety.

Furthermore, preference will be given to small or medium-sized breeds, as these are often perceived as less threatening to toddlers, thereby facilitating closer and safer interactions. This criterion seeks not only to reduce fear or discomfort but also to promote a relationship of trust and comfort between the toddler and the animal. It is equally important that the selected dog is accustomed to dynamic environments and varied stimuli, allowing it to adapt readily to sudden changes or unforeseen situations, responding in a calm and controlled manner. This approach ensures a positive experience for both the toddler and the animal, promoting the well-being and safety of both parties involved.

#### Robotic dog

The robot employed in this study is the TR-P5 Remote Controlled Dog, an advanced smart device designed to resemble a lifelike robotic puppy. It features voice recognition technology that enables communication via a remote control, allowing users to issue various vocal commands, such as “sit down," “stand up," perform “push-ups", and execute a “somersault". Additionally, it offers operational functions, including moving forward, moving backward, turning left, turning right, and dancing. This intelligent robotic dog is designed to respond in a manner akin to that of a real dog, enhancing user interaction. Furthermore, it is programmable, capable of executing up to 30 distinct actions, which facilitates the training of new commands.

### Data acquisition and analysis instruments

The primary components for data acquisition will include a high-definition camera for capturing data related to head tracking, while embedded devices equipped with accelerometers will be utilized to monitor movements and determine the dynamics of each activity. Additionally, for data analysis, software will be executed on a computer to detect sudden movements and monitor the status of the sensors S1 to S4. Further details regarding each of the components employed will be provided below.

#### Face-tracking camera

An OAK-D Pro camera [29] will be used to track the head movements and detect facial gestures of toddlers during interactions with the dog. The system will analyze these gestures to infer various emotional states, including drowsy, alert and inactive, alert and active, restless, and tearful. The camera combines high-definition RGB video and stereo depth sensing, providing accurate real-time data on head positioning and facial expressions. This data is critical for evaluating the toddlers’ attentional focus and emotional reactions. Additionally, the camera’s infrared capabilities allow it to function in various lighting conditions, ensuring consistent and reliable data collection. Its depth sensing also helps assess the toddler’s proximity to the dog, further enriching the analysis of their interaction.

#### Motion sensors

For tracking the movements of each limb, four ESP32-S3 Touch Display Development Boards [30] will be used, with one device placed on each of the toddler’s limbs (both arms and legs). Each of these boards comes equipped with an integrated accelerometer, which enables precise detection of movement, orientation, and dynamic motion patterns in real time. The accelerometer’s data will be collected to monitor the motion dynamics of the toddler’s limbs during the interaction with the dog or robotic animal.

The ESP32-S3 devices will leverage their built-in Wi-Fi capabilities to wirelessly transmit this motion data to a central processing system. This allows for the continuous tracking of limb movements without hindering the toddler’s natural range of motion. Each device will operate independently, sending real-time data that can be processed to evaluate motor coordination, movement patterns, and interaction dynamics.

**Fig 2:**
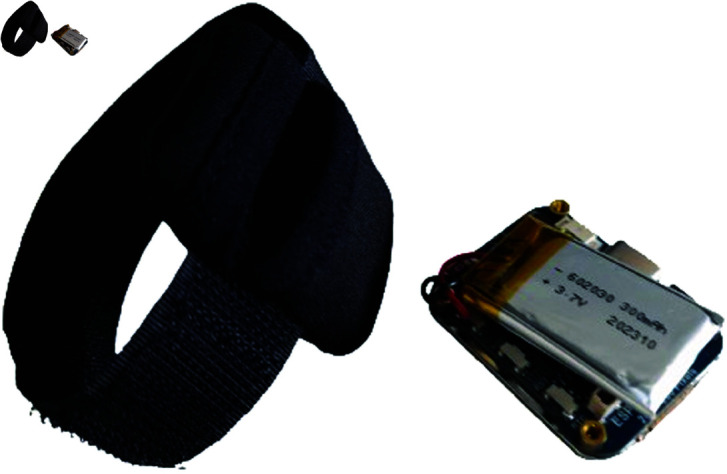
ESP32-S3 board with a battery for voltage supply and a wristband to attach to the infant’s limbs.

By distributing these devices across the four limbs, the setup ensures comprehensive motion tracking that can be analyzed to assess how the toddler engages physically with their environment during the experiment. Custom-made wristbands were developed for the sensors (see Fig 2), designed to be comfortable for toddlers while ensuring secure placement of the devices during the experiment.

#### Real-time monitoring software

A software program will run on a computer to monitor and collect the data transmitted by the sensors S1 to S4 in real-time, enabling the visualization of movement variations over time. The software will allow for continuous tracking of the toddler’s limb movements, providing insights into their dynamic interaction with the dogs. Additionally, it will process and analyze the data to detect any sudden or significant movements in each limb, which could indicate heightened attentiveness or emotional responses. These movements will be assessed in relation to the stimuli induced by the interaction with the dogs, helping to establish a direct correlation between physical actions and behavioral responses. The real-time monitoring feature will ensure that all data is captured promptly, facilitating immediate analysis and allowing for timely adjustments during the experimental process. This capability is crucial for understanding how toddlers react to the different interactions with the animals and enables researchers to track the evolution of their responses throughout the study.

## Detailed procedure

The infants will attend a laboratory setting for a single session lasting approximately 20 minutes. During this time, the baby will be placed in a chair with safety straps, and four motion sensors will be attached to their arms and legs. The proper transmission of the collected data will be verified to ensure accurate monitoring. Due to the age range of the participants and their varying heights, adjustments will be made to the camera position to ensure proper facial recognition of the infant. In the experimental setup, the caregiver was seated next to the infant but was instructed to refrain from any social interaction during the data collection process, except in cases where the infant is unable to tolerate the intervention and requires soothing. All study participants will be exposed to the interaction conditions listed in Table 3, during which the robotic dog and the real dog will behave as follows:

**Table 3:** Interaction condition performed by the robotic and real dogs for the intervention.

Interaction Cycle	Description
A1	The robotic dog greets by standing on two legs and barking.
A2	The robotic dog sits, barks, then stands up and barks again.
A3	The real dog plays by chasing its tail.
A4	The real dog plays by chasing a ball.

**Fig 3:**
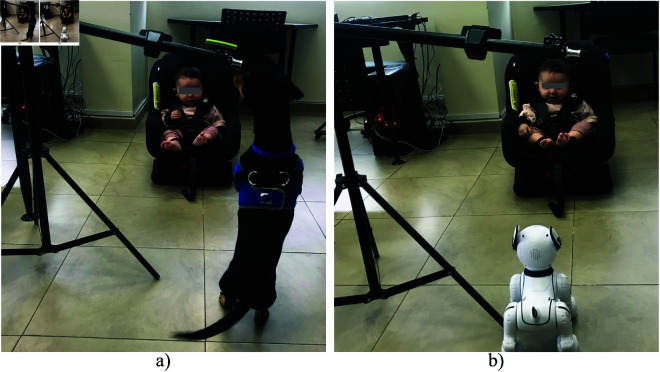
Experimental setup with a) real dog, and b) robotic dog.

A1 and A2 correspond to interaction actions between the robotic animal and the infant, which will be executed by the experiment operator using voice commands. A3 and A4 describe the actions performed by the dog, as directed by its trainer, in front of the infant. These actions will be conducted at a safe distance, ensuring the infant can observe without any risk of harm. Fig 3 illustrates an example of the experimental setup, showing interactions with both the real dog (a) and the robotic dog (b). The legal guardians of the individual in this manuscript has given written informed consent (as outlined in the PLOS consent form) to publish these case details.

**Fig 4:**
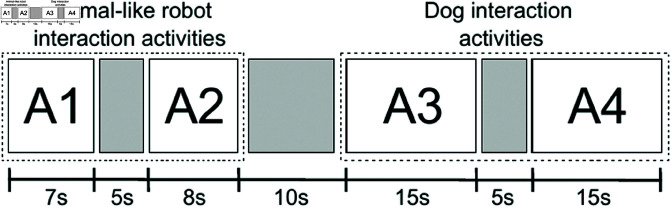
Interaction cycles. The gray squares correspond to inactive periods.

Fig 4 illustrates the duration of the four activities involved in the experiment, highlighting a 5-second period of inactivity between each task and a 10-second interval when transitioning from the actions performed by the robotic animal to those executed by the dog. Consequently, the total duration of the complete sequence is 65 seconds. This sequence will be repeated three times during each experimental trial with the infant. The aim is to analyze the data collected on facial gesture detection, head tracking, and limb activity to understand changes in the infant’s attention toward living versus non-living entities.

### Data analysis

Data analysis will be conducted using the statistical software SPSS, version 29, which provides robust and versatile tools for data handling. Initially, descriptive statistical analyses will be implemented with the aim of characterizing the sociodemographic information of the participants, as well as the results obtained from the various tests conducted. These analyses will include measures of central tendency, such as the mean and median, and measures of dispersion, such as standard deviation and range, allowing for a deeper understanding of the distribution and characteristics of the data.

Additionally, multivariate analysis of variance (MANOVA) will be conducted to evaluate significant differences between the experimental and control conditions across multiple dependent variables simultaneously. This approach will not only assess the overall impact of the interventions on participants but will also provide a comprehensive understanding of how the different experimental conditions influence the combined set of psychological outcomes. By considering the intercorrelations among the dependent variables, MANOVA enhances the precision of the analysis and offers a more robust evaluation of the interventions’ effectiveness.

Furthermore, correlation analyses, such as Pearson’s correlation coefficient, will be carried out to identify the quantitative association between the measurements taken. This approach will facilitate the exploration of significant relationships between variables and contribute to a more comprehensive understanding of how different factors interact within the context of attention in early childhood. Moreover, the possibility of conducting regression analyses will be considered, which will allow for the identification of significant predictors of outcomes and provide a more detailed insight into the underlying dynamics of the data.

## Expected results

The anticipated outcomes are grounded in well-established scientific principles linking physiological arousal and motor activity to attentional states in young children. It is expected that accelerometer data will provide quantifiable measures of movement patterns corresponding to varying levels of engagement. Based on the known association between atypical motor responses and attentional challenges, the protocol should effectively differentiate toddlers with normative attention profiles from those exhibiting early signs of difficulties. Furthermore, direct comparison of stimuli types (robotic vs. live dog) is predicted to yield measurable differences in motor responses, identifying the most effective attention-capturing stimulus for individual children.

To achieve these goals, this research aims to develop an innovative protocol for precise analysis of attentional processes in early childhood. The primary focus is designing a series of attention-stimulation activities evaluated through accelerometer sensors, which will quantify toddlers’ movement patterns and motor responses. By capturing these kinematic signatures, the sensors will objectively measure arousal levels (physiological activation) in response to distinct stimuli, thereby operationalizing attentional states into analyzable metrics.

Furthermore, establishing a robust protocol will not only measure attention levels in toddlers but also differentiate between those with low attention levels and those with optimal levels. This ability to discriminate between different degrees of attention will be crucial for the early identification of potential attentional disorders, such as attention deficit disorder, and will facilitate the implementation of personalized intervention processes.

In addition, the protocol will serve as a valuable tool for determining the effectiveness of different animated stimuli, allowing researchers to identify whether a robotic dog or a live dog better captures a toddler’s attention. This individualized analysis will enable the development of tailored strategies to enhance attentional engagement based on the toddler’s specific preferences.

In this way, this protocol will not only provide tools to enhance attention stimulation in toddlers but will also pave the way for preventive and therapeutic interventions that can be applied in the early stages of development. This will significantly contribute to the cognitive, emotional, and social well-being of toddlers by detecting and addressing potential attention issues before they affect their academic performance and overall development.

## Discussion

Conducting research on the attention of preschool children using accelerometer sensors and recognition cameras offers an innovative and objective perspective within this line of investigation. Accelerometers allow for the accurate capture of data regarding toddler’s physical activity, which can be correlated with their level of attention and concentration in various activities. On the other hand, recognition cameras can help observe visual behaviors and patterns of interest, providing additional information about how toddlers interact with their environment. This combination of technologies can enable a more comprehensive and detailed analysis of toddler behavior, facilitating the identification of patterns and trends that may be useful for developing educational strategies tailored to the needs of toddlers.

The findings of this study may offer significant benefits for the clinical management of attentional problems from an early age. A protocol of this nature could facilitate the early detection of attentional difficulties by comparing performance on specific levels of attention, such as focused attention, between children with and without cognitive impairments in this domain. Furthermore, the results could inform educational policies aimed at the early identification of attentional difficulties. Currently, referrals for neuropsychological assessment typically occur around the age of seven, once children have begun formal schooling. However, such evaluations should ideally take place earlier in development to provide timely support. The proposed protocol described in this article may serve as a valuable tool for identifying attention deficits during early childhood, thereby contributing meaningfully to interventions for children with attentional challenges.

Regarding the challenges to be considered in this study, research that utilizes accelerometer sensors and recognition cameras also faces several significant obstacles. The variability in toddler’s behavior can result in inconsistent data, as they are very active and can easily become distracted, making it difficult to establish a clear relationship between physical activity and attention. Additionally, the use of cameras raises ethical concerns related to toddler’s privacy, which may limit the feasibility of the study. It is also important to consider that these devices may not effectively capture the nuances of attention and concentration, which could complicate the interpretation of the results.

### Contribution to research in early human childhood

This study proposes to analyze the attention performance of toddlers in early childhood using accelerometer sensors and recognition cameras, representing a significant contribution to the research line on attention during this developmental stage. By employing advanced technologies, such as accelerometers, it is possible to measure the physical activity and body movements of toddlers, providing data on their attention levels and how these relate to their behavior. On the other hand, the use of recognition cameras allows for the observation and recording of visual patterns and social interactions, which is crucial for understanding how toddlers focus their attention on different stimuli in their environment. This multidimensional approach not only enables a more objective and accurate assessment of attentional performance but also facilitates the identification of potential attentional difficulties that could influence toddler’s learning and emotional development. Furthermore, the findings of this study could guide the creation of early and personalized interventions aimed at improving toddler’s attention and well-being, thereby contributing to the development of more effective and evidence-based educational practices. Ultimately, this type of research not only enriches academic knowledge about attention in childhood but also has the potential to positively impact the formulation of policies and programs directed at education and child health.
